# Significance of Microalbuminuria in Predicting Silent Myocardial Ischemia in Patients with Type 2 Diabetes Using Myocardial Perfusion Imaging

**DOI:** 10.4274/mirt.galenos.2019.93798

**Published:** 2019-06-24

**Authors:** Tayyebeh Emami, Zohreh Naeimei, Azita Salehifard, Zahra Azizmohammadi, Dariush Iranpour, Mohammadreza Kalantarhormozi, Esmail Jafari, Ali Gholamrezanezhad, Majid Assadi

**Affiliations:** 1 Bushehr University of Medical Sciences, School of Medicine, Bushehr Medical University Hospital, Department of Internal Medicine, Division of Endocrine Disorders, Bushehr, Iran; 2 Shahid Beheshti University of Medical Sciences, Imam Hossein Hospital, Department of Nuclear Medicine, Tehran, Iran; 3 Bushehr University of Medical Sciences, School of Medicine, Bushehr Medical University Hospital, Department of Cardiology, Bushehr, Iran; 4 Bushehr University of Medical Sciences, Bushehr Medical University Hospital, The Persian Gulf Nuclear Medicine Research Center, Department of Molecular Imaging and Radionuclide Therapy, Bushehr, Iran; 5 University of Southern California, Keck School of Medicine, Department of Diagnostic Radiology, Los Angeles, USA

**Keywords:** Microalbuminuria, silent myocardial ischemia, type 2 diabetes, myocardial perfusion imaging

## Abstract

**Objectives:**

In light of increased risk of cardiovascular events and the poor prognosis of coronary artery disease (CAD) in diabetic versus non-diabetic patients and also with respect to the importance of early diagnosis of CAD in this status, the study was aimed to assess the importance of microalbuminuria in predicting silent myocardial ischemia (SMI) in patients with type 2 diabetes using myocardial perfusion imaging (MPI).

**Methods:**

This study included 120 patients with diabetes type 2, but without previously known CAD or any cardiac symptoms that were stratified into two groups based on presence/absence of microalbuminuria. All participants underwent CAD evaluation using gated myocardial perfusion single-photon emission computed tomography (MPS) imaging. Other clinical and laboratory indices were also recorded.

**Results:**

Studied population consisted of 84 males (70%) and 36 females (30%), totally 120 patients with mean age of 58.61±9.90). In total, asymptomatic ischemia was detected in 78 (65%) of the included diabetic patients. Stress induced ischemia was found in 56 patients (87.5%) of albumin+ (Alb) group and in 22 patients (39.3%) of Alb- group. The frequency of stress induced ischemia was 10.81 times higher in the patients with microalbuminuria compared to Alb- ones [p<0.001, Odds ratio: 10.81, 95% confidence interval: 4.33-26.99]. On the other hand, no relationship was found between the presence of stress induced ischemia and therapy type, diabetes duration, history of evident retinopathy, history of hypertension and also serum levels of hemoglobin A1c (p>0.05).

**Conclusion:**

The current study showed that abnormal MPI findings are significantly more common in diabetic patients with microalbuminuria. With respect to low cost and availability of urine Alb detection tests, it might be as a biomarker for prediction of SMI in daibetic population.

## Abstract (tr)

**Amaç:**

Bu çalışmada diyabetik olgularda diyabetik olmayanlara göre kardiyovasküler olay riskinin yüksek ve koroner arter hastalığı (CAD) prognozunun kötü olmasından yola çıkarak tip 2 diyabette CAD’nin erken tanısında mikroalbuminürinin önemi miyokard perfüzyon sintigrafisinde (MPS) saptanan sessiz miyokard iskemisi (SMİ) bulguları ile birlikte değerlendirilmektedir.

**Yöntem:**

Önceden bilinen CAD ve kardiyak semptomu bulunmayan tip 2 diyabetli 120 olgu çalışmaya dahil edilerek mikroalbuminüri varlığına/yokluğuna göre iki gruba ayrılarak incelendi. Tüm hastalara CAD’yi değerlendirmek üzere miyokard perfüzyon tek-foton emisyonlu bilgisayarlı tomografi ile görüntüleme yapıldı. Ayrıca diğer klinik ve laboratuvar verileri toplandı.

**Bulgular:**

Çalışma grubu 84 erkek (%70) ve 36 kadından (%30) oluşan, yaş ortalaması 58,61±9,90 olan 120 hastalık bir gruptur. Gruba dahil edilen diyabetik olguların 78’inde (%65) asemptomatik iskemi saptandı. Albumin+ (Alb) olguların 56’sında (%87,5), Alb- olguların 22’sinde (%39,3) stres iskemisi bulundu. Mikroalbuminüri bulunan olgularda stres iskemisi sıklığı diğerlerine göre 10,81 kez fazlaydı [p<0,001, Odds oranı: 10,81, %95 güven aralığı: 4,33-26,99]. Öte yandan, stres iskemisi ile tedavi tipi, diyabetin süresi, bariz retinopati öyküsü, hipertansiyon ve serum hemoglobin A1c düzeyi arasında ilişki bulunmadı (p>0,05).

**Sonuç:**

Bu çalışma mikroalbuminürisi bulunan diyabetik olgularda MPS’sinde anormal bulguların daha sık olduğunu göstermektedir. Düşük maliyeti ve kolay temini nedeniyle idrarda albumin ölçümü diyabetik popülasyonda SMİ’yi öngörmede bir biyobelirteç olarak kullanılabilir.

## Introduction

Type 2 diabetes is defined as impairment of the ability to produce or respond to the insulin hormone, leading to abnormal metabolism of carbohydrates and increasing in levels of blood glucose. The risk of type 2 diabetes increases with age specifically over 50 years old.

Globally about 400 million adults are living with diabetes mellitus around the world and it is predicted that this number will be increased to more than 640 million until year 2040 (1).

About 4 million deaths per year are attributable to diabetes side effects, which is 9% of all deaths worldwide. These side effects include cardiovascular and cerebrovascular attacks, retinopathy, nephropathy, neuropathy and non-traumatic limbs amputations (2,3,4,5).

Cardiovascular events including coronary artery disease (CAD) 40% and other cardiac disorders like chronic heart failure 15% are the leading cause of morbidity and mortality of patients with diabetes, and death will occur 14.6 years earlier in type 2 diabetic patients compared to non-diabetics (5).

Microalbuminuria, defined as urinary albumin excretion of 20-200 mg/day, is a marker of systemic vascular damage, renal functional impairment and CAD (2).The prevalence of microalbuminuria is estimated 19% in diabetic patientsas a marker of renal, cardiac and cerebral vascular damage (6).

Clinically patients with diabetes are more likely to be without chest pain in the setting of unstable angina, myocardial infarction or during exercise testing, and thus late presentation contributes to late CAD diagnosis and a higher mortality in these patients (7).

Silent myocardial ischemia (SMI) is classically described as an objective document of myocardial ischemia in patients without subjective ischemia symptoms. Now, there are different clinical methods in the diagnostic evaluation of CAD. Coronary artery angiography (CAG) is the gold standard for distinguishing of asymptomatic CAD. Computed tomography coronary angiography (CTCA) can depict anatomy, trend and extent of coronary stenosis. Myocardial perfusion imaging (MPI) uses to diagnose whether anatomical stenosis yields to myocardial dysfunction, to assess the risk estimation and prognosis of myocardial disease and also is frequently applied in clinical evaluation of CAD (8,9,10,11,12,13).

In view of increased risk of cardiovascular events and the poor prognosis of CAD in diabetic versus non-diabetic patients and also with respect to the importance of early diagnosis of CAD in this status, the study was aimed to assess the importance of microalbuminuria in predicting SMI in patients with type 2 diabetes using MPI.

## Material and Methods

### Study Population

Our study was designed as a non-randomized cross sectional clinical study. The study evaluated 120 patients with known and established diabetes type 2, but without previously known CAD or any cardiac symptoms.

The patients with past history of acute coronary syndrome, myocardial ischemia, abnormal electrocardiogram, previous myocardial infarction, percutaneous CTCA, coronary artery bypass graft surgery, peripheral vascular disease, established predisposing malignancy, chronic inflammatory disorders (vasculitis, rheumatoid arthritis, systemic lupus erythematosus), severe systemic illnesses and renal diseases were excluded from the study.

Evaluation for CAD was performed using MPI gated single-photon emission computed tomography (SPECT) imaging in the department of nuclear medicine of a university affiliated hospital. MPI findings were compared to the microalbuminuria status. Other clinical indices like presence of retinopathy, hypertension, systolic and diastolic blood pressure, liver function tests (aspartate aminotransferase, alanine aminotransferase, alkaline phosphatase), serum lipid profile (high density lipoprotein, low density lipoprotein, thyroglobulin), hemoglobin A1c (HbA1c) and blood urea nitrogen/serum creatinine were also recorded. It should be mentioned that all eligible patients signed an inform consent. This study complies with the Declaration of Helsinki, and it was approved by the Institutional Ethics Committee of Bushehr University of Medical Sciences.

**Microalbuminuria Assessment:** Microalbuminuria was measured after 24 h urine collection and the patients were divided into two groups with microalbuminuria [renal albumin excretion between 20-200 mg/day, albumin^+ ^(Alb)] and without microalbuminuria (renal albumin excretion less than 20 mg/day, Alb^-^). The patients with more than 200 mg/day were excluded.

**Gated-SPECT MPI: **Patients were instructed to refrain from caffeine-containing beverages for at least 12 hours, nitrates for 24 hours and beta-blockers for 48 hours before the study.

A two-day stress/rest MPI protocol was carried out for all patients. At stress phase, weight –adjusted doses of 10 MBq/kg of Tc-99m methoxyisobutylisonitrile (at least 700 MBq) was injected at peak pharmacologic stress and the similar dose was injected at rest for each patient on the next day.

Pharmacologic stress was obtained by 0.56 mg/kg of body weight of dipyridamole in 20 mLnormal saline which was injected intravenously during 5 minutes under electrocardiographic monitoring. Four minutes later, Tc-99m MIBI was injected.

SPECT acquisition was performed almost 30 minutes later. At the rest phase, almost 45 minutes after injection of Tc-99m MIBI, SPECT acquisition was performed for each patient.

**SPECT Imaging Protocol**: Images were done over a 180º orbit from right anterior oblique 45º to left posterior oblique 45º using a dual-head γ-camera (ADAC, USA) equipped with ultra-high resolution collimator. Acquisition was carried out in 32 steps at 30 seconds per stepusing the step- acquisition mode. For image acquisition, a 20% acceptance window around the 140 keV photopeak was applied. A 64x64 matrix was applied for all acquisitions. The prefilteration of projection datasets was performed by a Butterworth filter and images were reconstructed by filtered back-projection. For all images, a technologist experienced in nuclear cardiology reconstructed the raw data.

**Interpretation of MPIs: **All images were assessed qualitatively by two experienced  practitioners,  who reached an agreement on the results. The physicians were blind to the patient’s data. Segmental perfusion defect in the stress phase images, which revealed filling-in (more uptake) in the rest phase study was considered as ischemia or reversible perfusion defect. Segment with perfusion defect in the stress phase scan with no alteration in size or amount of uptake (perfusion score) in the rest phase images were considered as irreversible perfusion defect or scar tissue.

### Statistical Analysis

The continuous variables are presented as the mean ± standard deviation, and categorical variables as the absolute values and percentages. Categorical variables were compared using chi-square test and continuous variables using unpaired Student’s t-test. Statistical analysis was performed with the use of the SPSS Statistical Package (version 20). A p value <0.05 was considered statistically significant.

## Results

Studied population consisted of 84 males (70%) and 36 females (30%), totally 120 patients with mean age of 58.61±9.90 years old. In terms of duration of diabetes, 39 patients (32.5%) had more than 10 years, 39 patients (32.5%) had between 5-10 years and 42 patients (35%) had less than five years diabetes history. Anti-diabetic therapy used by the patients were as follow: 44 patients (36.7%) metformin, 33 patients (27.5%) metformin and glibenclamide combination, seven patients (5.8%) glibenclamide, six patients (5%) metformin and insulin combination, three patients (2.5%) insulin and one patient (0.8%) metformin, glibenclamide and insulin combination (Table 1). In addition, 87 patients were using aspirin. The type of antidiabetic therapy in our participants, did not show any significance in neither group Alb^+^ nor Alb^-^ (p>0.05).

Totally, 64 patients (53.3%) had the history of microalbuminuria, 30 patients (25%) had the history of established retinopathy and 76 patients (63.3%) had the history of hypertension.

**Comparison of Diabetic Patients with and without Microalbuminuria: **From 64 patients with established microalbuminuria (group Alb^+^), 25 (39.1%) were male and 39 (60.9%) were female, and from 56 patients without evident microalbuminuria (group Alb^-^), 11 (19.6%) and 45 (80.4%) were male and female respectively. The abundance of microalbuminuria incidence was 2.62 times higher in males compared to female patients [Odds ratio (OR): 2.62, 95% confidence interval (CI): 1.14-6.00, p=0.02].

Average age of the patients with and without microalbuminuria was 59.37±9.42 years old and 57.75±10.43 years old, respectively which showed no statistical significance (p=0.37).

From 64 patients in group Alb^+^, 41 (64.1%) and 23 (35.9%) cases had evident diabetes more and less than five years, respectively. Likewise, from 56 patients in group Alb^-^, 25 (44.6%) and 31 (55.4%) cases had evident diabetes more and less than five years, respectively.

Among all diabetic patients in our study, the risk of developing microalbuminuria was 2.21 times higher in patients with the history of over five years diabetes disease compared to the patients with the history of less than five years evident diabetes (OR: 2.21, 95% CI: 1.06-4.60, p=0.03).

The average of the duration of diagnosed diabetes were 9.21±6.56 years in group Alb^+^ and 6.66±4.84 years in group Alb^-^ and showed statistically significance (p=0.01).

Among the patients with microalbuminuria, 20 (31.3%) and among the patients without microalbuminuria, 10 (17.9%) had the history of retinal vascular surgery but it didn’t show statistically significance (p=0.09).

Likewise, in group Alb^+^, 41(64.1%) patients and in group Alb^-^, 35 (62.5%) patients had the history of diagnosed hypertension (p=0.85).

The average of systolic and diastolic blood pressures was 126.17±4.77 mmHg and 84.68±4.16 mmHg, as well as in group Alb^+^, 125.98±4.90 mmHg and 84.10±4.27 mmHg in group Alb^-^ (p=0.83, p=0.45), respectively.

Laboratory test results are shown in Table 2 and showed no significance between group Alb^+^ and Alb^-^ (p>0.05).

**Myocardial Gated SPECT Findings:** In total, SMI was detected in 78 (65%) of the included diabetic patients. Stress induced ischemia was found in 56 patients (87.5%) of Alb^+^ group and in 22 patients (39.3%) of Alb^- ^group. As described, the frequency of stress induced ischemia was 10.81 times higher in the patients with microalbuminuria compared to Alb^-^ patients (p<0.001, OR: 10.81, 95% CI: 4.33-26.99). Likewise, this ischemic status was also correlated to the gender and occurred 2.95 times more in males than females. From 36 male cases, 29 (80.55%) and from 84 female cases, only 49 (58.33%) showed stress induced myocardial ischemia (OR: 2.95, 95% CI: 1.16-7.51, p=0.019). No correlation was found among the presence of stress induced ischemia and therapy type, diabetes duration, history of evident retinopathy, history of hypertension and serum levels of HbA1c (p>0.05). In addition, cardiac SPECT findings revealed presence of septal hypertrophy in 40 patients (62.5%) of group Alb^+^ and in 37 patients (66.1%) of group Alb^-^ without any statistically significance (p=0.684).

Average ejection fraction (FE) was estimated 53.51±3.17% for Alb^+^ patients and 56.07±3.77% for Alb^-^ ones and EF was significantly higher in Alb^-^ patients compared to the other group (p=0.001) (Table 3).

## Discussion

Diabetes mellitus is one of the major disabling diseases around the world. Prevalence and incidence of type 2 diabetes is increasing over time especially in under developed countries. Increasing diabetes prevalence will lead to increasing the side effects of this disease as well as patient’s morbidity and mortality. Diabetes is accompanied with 2 to 4 times increased risk of the CAD development and progression (14). The mortality resulted from any cause including CAD likely will be more in younger patients who have also higher serum glucose levels and suffer from diabetic nephropathy (15,16).

SMI is the most common manifestation of CAD in diabetic patients and can be manifested as myocardial infarction or death in some patients. The reported prevalence of SMI in diabetic patients ranges between 6 to 57% (17,18), but it was estimated 65% in our recent study which could be related to the use of different methodologies through the studies. Diabetic neuropathy is the most common underlying cause of silent ischemia in about 22-42% of asymptomatic diabetic patients (19,20,21). Furthermore, about 19% of diabetics have renal protein excretion and most studies found the increased risk of cardiovascular events in the patients with overt microalbuminuria (22,23). Microalbuminuria is the first sign of renal involvement and incoming diabetic nephropathy (10), likewise it could be taken as an accurate and sensitive predictive indicator of end stage renal disease in diabetic patients (6). Microalbuminuria is also a marker of cardiac and cerebral vascular damage in addition to renal damage (2).

Accordingly, this study was designed to show the correlation between microalbuminuria and the presence of CAD in asymptomatic diabetic patients by evaluation of myocardial perfusion scan findings. Our study showed that, although the duration of diabetes is not correlated to patient’s gender, the frequency of the development of microalbuminuria is 2.62 times higher in males. Probability of microalbuminuria development in diabetic patients was 2.21 times higher in patients with the history of diabetic disease more than five years.

In the evaluation of MPI findings, average FE was lower in patients with microalbuminuria and also prevalence of ischemia was 10.81 times higher than the patients without microalbuminuria. This result is contrary to the results of The Detection of Ischemia in Asymptomatic Diabetics (DIAD) study by Wackers et al. (18), which showed no significant association between microalbuminuria and perfusion defects on myocardial perfusion scan.

Ischemia was also correlated with patient’s gender and we found ischemia 2.95 times more in males than females as in DIAD study by Wackers et al. (18). We also found no association between myocardial ischemia and therapy type, diabetes duration, history of retinopathy, history of hypertension and serum levels of HbA1c.

Salehi et al. (11) evaluated the MPI findings in diabetic patients, and they found a relationship between diabetes duration and abnormal MPI findings, so that patients with longer diabetes duration showed 2.27 times more MPI abnormalities. These results were contrary to our findings. They indicated that performing myocardial perfusion SPECT in asymptomatic diabetic patients will lead to early CAD diagnosis and should be considered as a screening tool in cases with diabetes.

Shmendi et al. (24) evaluated the findings of myocardial perfusion scan in diabetic patients with a suspicion of myocardial ischemia. They found that abnormal MPI findings, including stress inducible ischemia, were seen more in diabetic patients compared to non-diabetics. On the other hand, HbA1c >7% was related to more abnormal myocardial perfusion SPECT (MPS) findings and ischemia risk in diabetic patients. The results of this study are not in agreement with our current results as we did not find any correlation between myocardial ischemia and HbA1c level >7%. Finally, they showed that the frequency of abnormal MPS findings and myocardial ischemia is higher in diabetic patients versus non-diabetics. Likewise poorer control of serum glucose level resulted more probability of ischemia in diabetics.

In another study, Al-Humaidi et al. (10) worked on myocardial perfusion scan abnormalities in asymptomatic patients with type 2 diabetes. They found abnormal MPI findings in 22 (37%) of 59 patients. In their study, abnormal MPI was found to be correlated well with diabetes duration, insulin therapy, diabetic nephropathy and neuropathy. However in the current study, only gender and microalbuminuria were correlated with abnormal MPS results. They also represent that abnormal MPI results are more prevalent in asymptomatic diabetic patients and they should be screened with MPI if they have high CAD pre-test probability.

Potier et al. (14) assessed the correlation between cardiac microvascular dysfunction and microalbuminuria in diabetics with ^82^Rubidium-positron-emission tomography scan. In their study, myocardial flow reserve (MFR) was significantly lower in diabetic patients versus non-diabetics. On the other hand, MFR was progressively declined parallel to increasing albumin secretion in urine. Whereas MFR as a marker of myocardial ischemia, the results of their study are consistent with our results. MFR was not significantly different in patients with or without retinopathy but micro and macroalbuminuria was associated with abnormal MFR. They finally emphasized that abnormal MFR is strongly related to diabetes and the severity of albumin secretion in urine.

In a study by Giovacchini et al. (25) the frequency of CAD in diabetic patients was evaluated and in similar to our study, their results showed that microalbuminuria is the only predicting factor for silent ischemia in asymptomatic diabetic patients and the incidence of ischemia is 4.42 times higher in patients with microalbuminuria.

Furthermore, it has been shown that microalbuminuria and left ventricular hypertrophy are both associated with increased cardiovascular morbidity and mortality, especially in diabetic patients. Therefore, it has been recommended that patients with type 2 diabetes and increased urinary albumin excretion should be check for increased left ventricular mass as an important and potentially reversible cardiovascular risk factor (26,27,28).

Additionally, it has been demonstrated that increased septal perfusion observed on MPI is the signal of asymmetrical septal hypertrophy which can be graded based on its severity (29). In our study, although the septal hypertrophy was shown in 76 patients, it was not statistically significant between two Alb^+^ and Alb^-^ groups (p value >0.05).

### Study Limitations

It should also be mentioned that our study has some limitations. The most important limitation is the lack of follow-up to assess the patient’s clinical outcome; although in our prior experience, about 50% of the patients with abnormal MPI findings demonstrated abnormal CAG (13) and also should be considered the point that CAG does not reflect myocardial perfusion at the terminal coronary circulation and in cases with SMI, false negative findings may happen. The small sample size and lack of quantitative evaluation of myocardial ischemia on MPS are other limitations that should be underlined. Further well-designed studies with large number of patients using quantitative analysis of myocardial perfusion SPECT will be required to validate its clinical role.

## Conclusion

The current study showed that abnormal MPI findings are significantly more common in diabetic patients with microalbuminuria. With respect to low cost and availability of urine albumin detection tests, it might be as a biomarker for prediction of SMI in diabetic population.

## Figures and Tables

**Table 1:**
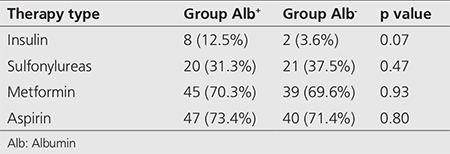
Patient’s data according to the use of antidiabetic medication

**Table 2:**
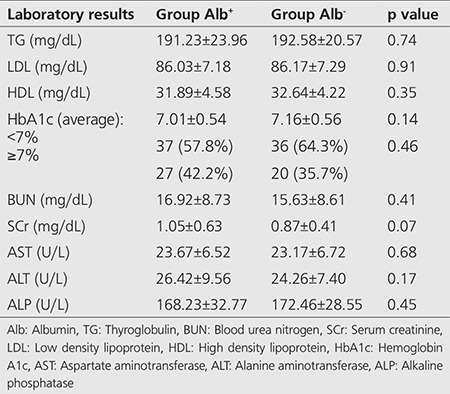
The presentation of overall laboratory tests

**Table 3:**
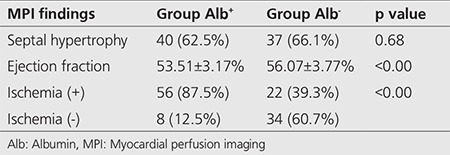
Myocardial perfusion single-photon emission computed tomography findings
